# Predicted Glycerol 3-Phosphate Dehydrogenase Homologs and the Glycerol Kinase GlcA Coordinately Adapt to Various Carbon Sources and Osmotic Stress in *Aspergillus fumigatus*

**DOI:** 10.1534/g3.118.200253

**Published:** 2018-05-08

**Authors:** Chi Zhang, Xiuhua Meng, Huiyu Gu, Zhihua Ma, Ling Lu

**Affiliations:** Jiangsu Key Laboratory for Microbes and Functional Genomics, Jiangsu Engineering and Technology Research Center for Microbiology, College of Life Sciences, Nanjing Normal University, Nanjing, 210023, China

**Keywords:** *Aspergillus fumigatus*, glycerol 3-phosphate dehydrogenase, stress, high osmolarity glycerol (HOG) pathway

## Abstract

Glycerol plays an important role in the adaptation of fungi to various microenvironments and stressors, including heat shock, anoxic conditions and osmotic stress. Glycerol 3-phosphate dehydrogenase (G3PDH) is able to catalyze dihydroxyacetone phosphate to glycerol 3-phosphate (G3P), which is subsequently dephosphorylated into glycerol. However, current knowledge about the functions of G3PDH homologs in glycerol biosynthesis in *Aspergillus fumigatus* is limited. Here, we show that the *A. fumigatus* G3PDH gene, *gfdA*, is crucial for normal colony growth in glucose media under both normoxic and hypoxic conditions. In addition, failure of the overexpression of the *gfdA* homolog, *gfdB*, to rescue the phenotype of a *gfdA* null mutant suggests that *gfdA* plays a predominant role in the synthesis of G3P and glycerol. However, in a wild-type background, overexpressing either *gfdA* or *gfdB* is able to significantly enhance biomass production of mycelia, suggesting that *gfdA* and *gfdB* have similar functions in promoting the use of glucose. Interestingly, overexpression of the gene encoding the predicted glycerol kinase, GlcA, which is capable of phosphorylating glycerol to form G3P, significantly rescues the growth defects of *gfdA* null mutants in glucose media, indicating that the growth defects of *gfdA* null mutants might be due to the absence of G3P rather than glycerol. Moreover, Western blotting analysis revealed that *gfdA* is inducibly expressed by osmotic mediators. However, in the absence of *gfdA*, osmotic stress can rescue colony growth defects and allow colonies to partially bypass the *gfdA* requirement in a high osmolarity glycerol pathway-dependent manner. Therefore, the findings of this study elucidate how saprophytic filamentous fungi have developed pathways distinct from those of budding yeasts to adapt to varied carbon sources and survive environmental stresses.

Rapid adaptation responses to heat shock, anoxic conditions, and osmotic stresses, are crucial for the survival and proliferation of environmental fungi (Ansell *et al.* 1997; Aldiguier *et al.* 2004; Valadi *et al.* 2004; Boyce *et al.* 2016; Brown and Goldman 2016). To adapt to external stress conditions, fungi activate intracellular signaling systems to generate different metabolites (Pahlman *et al.* 2001; Kojima *et al.* 2004; Brown and Goldman 2016; Pereira Silva *et al.* 2017). In the model yeast *Saccharomyces cerevisiae*, glycerol, an important metabolite and osmolyte, is synthesized, accumulated and retained in cells in response to multiple external stresses (Rep *et al.* 1999; Siderius *et al.* 2000; Pahlman *et al.* 2001; Lee *et al.* 2012; Aslankoohi *et al.* 2015). Glycerol is also a precursor of phospholipids and helps maintain cellular redox balance (Maeda *et al.* 1994; Ansell *et al.* 1997; Siderius *et al.* 2000). Previous studies have reported that the glycerol synthesis pathway of the model filamentous fungus *Aspergillus nidulans* is similar to that of *S. cerevisiae* (Arst *et al.* 1990; Norbeck and Blomberg 1997; Fillinger *et al.* 2001). In this pathway, the glycolytic intermediate dihydroxyacetone phosphate (DHAP) is first reduced to glycerol 3-phosphate (G3P), a reaction catalyzed by NAD-dependent glycerol 3-phosphate dehydrogenase (G3PDH), and then G3P is dephosphorylated into glycerol by specific phosphatases. Alternatively, glycerol can be synthesized by another pathway in which the glycolytic intermediate DHAP is dephosphorylated to dihydroxyacetone (DHA), which is followed by its reduction to glycerol (Siderius *et al.* 2000).

In different *S. cerevisiae* strain backgrounds, G3PDHs encoded by *GPD1* and *GPD2* belong to a group of rate-limiting enzymes that control glycerol synthesis under a variety of media and oxygenation conditions. Although lack of both *GPD1* and *GPD2* leads to the complete loss of glycerol formation under all tested conditions, loss of either *GPD1* or *GPD2* does not generate a noticeable change in the glycerol yield (Nissen *et al.* 2000; Hubmann *et al.* 2011). Previous studies have also shown that *GPD1* plays a crucial role in osmotic adaptation, since the *GPD1* mutant shows hypersensitivity to osmotic stress and the expression of *GPD1* is induced by external osmotic stress (Albertyn *et al.* 1994). In contrast, the expression of *GPD2* is not affected by elevated external osmotic stress, but is induced in response to anoxic stress. The *GPD2* null mutant also grows poorly in an anoxic environment. A double *GPD1, GPD2* mutant is highly sensitive to both osmolarity and anoxia (Guadalupe Medina *et al.* 2010). Further studies have shown that the increased transcript responses of *GPD1* triggered by extracellular osmotic stress are regulated by the high osmolarity glycerol (HOG) pathway, which is the central signal transduction system the osmotic stress response (Albertyn *et al.* 1994). Unlike the activation of *GPD1*, the hypoxic activation of *GPD2* transcription is reported to be independent of the HOG pathway. Instead, it is controlled by another oxygen-independent signaling pathway (Eriksson *et al.* 1995; Ansell *et al.* 1997). In comparison, the model fungus *A. nidulans* also possesses two G3PDH-encoding genes, *gfdA* and *gfdB*. The expression of *gfdA* from *A. nidulans* fully rescues the growth defects of the *S. cerevisiae GPD1* null mutant under osmotic stress, suggesting that *gfdA* and *GPD1* have conserved functions. The *gfdA* null mutant exhibits reduced intracellular G3P levels and osmo-remediable defects on various carbon sources,with the exception of glycerol. In contrast, the functions of *gfdB* have not been explored in any species of *Aspergillus* (Fillinger *et al.* 2001; Furukawa *et al.* 2007).

As a saprophytic fungus with a large number of buoyant airborne conidia, *Aspergillus fumigatus* is ubiquitously present in the environment due to its rapid adaptation to different carbon and nitrogen sources, heat shock, anoxic conditions, osmotic stress and other environmental stressors (Brown and Goldman 2016; Bruder Nascimento *et al.* 2016; Ries *et al.* 2017). However, knowledge of how the glycerol pathway genes participate in stress responses and adaptation to varied carbon resources is limited in this medically important human pathogen. In this study, we show that *gfdA* of *A. fumigatus* is required for normal colony growth in glucose media under both normoxia and hypoxia and that the overexpression of a predicted glycerol kinase, GlcA, which phosphorylates glycerol to G3P, is able to significantly rescue the growth defects of a *gfdA* null mutant in glucose medium. Our findings indicate that, compared to the model organism *S. cerevisiae*, the opportunistic human fungal pathogen *A. fumigatus* has developed a unique glycerol biosynthesis network to adapt to various carbon sources and respond to osmotic stress.

## Materials and Methods

### Strains, media, and culture conditions

A list of *A. fumigatus* strains used in this study is provided in the supplementary data Table S1. Strains were grown in the following media: glucose media (minimal media) containing 1% glucose, 2% agar, 1 mL/L trace elements and 50 mL/L 20 × salt solution, as described previously (Zhang and Lu 2017); glycerol media containing 1% (10 mL/L) glycerol, 2% agar, 1 mL/L trace elements and 50 mL/L 20 × salt solution; Rich media (YAG) containing 0.5% yeast extract, 2% agar, 2% glucose and 1 mL/L trace elements; YUU (for uracil and uridine auxotrophic strains) media containing YAG, 5 mM uridine and 10 mM uracil. Liquid media were identical to the corresponding solid agar media, except for the omission of agar. All strains were cultured at 37° under the normoxic or hypoxic conditions. The hypoxic condition was established in 250 mL sealed bags containing an AnaeroPack-Anaero (Mitsubishi Gas Chemical Company), a disposable oxygen-absorbing and carbon dioxide-generating agent used in anaerobic pouches.

### Constructions for GFP labeling and deletion strains

The *gfdA-GFP* strain was constructed using the MMEJ-CRISPR system as described previously (Zhang *et al.* 2016a; Zhang and Lu 2017). The sgRNA, which targets the terminator site of *gfdA*, was synthesized *in vitro* by MEGAscript T7 Kit (Life Technologies, cat. no. AM1333).The corresponding repair template, including a fragment of GFP-hph (*hph* is a hygromycin selectable marker) with microhomology arms, was amplified by PCR. Then, fusion products of GFP-hph and sgRNA were cotransformed into a Cas9-expressing *A. fumigatus* recipient strain. For gene deletions, a similar strategy was carried out, but using sgRNA targeted to the open reading frame (ORF) of the target gene. The primers and annotations for sgRNAs and repair templates are listed in Table S2.

### Constructs for complementation and overexpression assays

The plasmid (p-zero-pyr4-gfdA) for *gfdA* complementation was generated as follows: the selectable marker *pyr4* from *Neurospora crassa* was amplified by PCR using the primers pyr4-*Spe*I-F and pyr4-*Spe*I-R and then cloned into the the pEASY-Blunt vector (TransGen Biotech) generating a plasmid P-zero-pyr4. Primers gfdA-revertant-F and gfdA-revertant-R were used to generate a fragment that includes the promoter sequence, the complete ORF, and the 3′UTR of *gfdA*. This fragment was then cloned into the *Not*I site of plasmid P-zero-pyr4 to generate the p-zero-pry4-gfdA plasmid.

For construction of the overexpression strains, plasmids overexpressing *gfdA*, *gfdB*, or *glcA* were generated as follows: PCR, using the primers gpd-gfdA/gfdB/glcA-ATG-F and gfdA/gfdB/glcA-*Bam*HI-R, was used to generate fragments that included the complete ORF and 3′UTR of relative indicated genes. Next, the fusion fragments gpdA-gfdA, gpdA-gfdB, or gpdA-glcA, which included gpdA (promoter sequence amplified with gpd-*Bam*HI-F and gpd-down) and its relative gene amplified with primers gpd-*Bam*HI-F and gfdA/gfdB/glcA-*Bam*HI-R, were generated by fusion PCR, respectively. The three fusion fragments were, respectively, subcloned into the *Bam*HI site of the plasmid prg3-AMAI-*Not*I to generate overexpression plasmids pAMAI-gpd-gfdA, pAMAI-gpd-gfdB, and pAMAI-gpd-glcA, which contain the *pyr4* marker (Aleksenko and Clutterbuck 1997). Transformation procedures were carried out as previously described (Zhang and Lu 2017). Transformants were selected in the medium without uridine and uracil or in the presence of 150 μg/mL hygromycin B (Sangon) or 0.1 μg/mL pyrithiamine (Sigma). In order to recycle the *pyr4* selectable marker, 5-FOA resistance (1 mg/mL 5-FOA) was selected in the recipient strains. All primers used are listed in the supplementary data Table S2.

### Strain verification by diagnostic PCR and Southern blotting

All transformant isolates were verified by diagnostic PCR analysis using mycelia as the source of DNA. Primers were designed to hybridize upstream and downstream of the expected cleavage sites as labeled in Figure S1. For Southern blotting, genomic DNA was digested with *Bam*HI, separated by electrophoresis, and transferred to a nylon membrane. A 0.7 kb fragment amplified with primers gfdA/gfdBprobeF and gfdA/gfdBprobeR was used as a probe. Labeling and visualization were performed using a DIG DNA labeling and detection kit (Roche Applied Science), according to the manufacturer’s instructions. The details are given in the supplementary data Figure S1.

### Western blotting

To extract proteins from *A. fumigatus* mycelia, 10^8^ conidia were inoculated in liquid glucose media with or without 1M sorbitol/NaCl at 220 rpm on a rotary shaker at 37° for 24 h. Protein extraction was performed as previously described (Nandakumar *et al.* 2003). Western blotting was performed as routine procedures (Zhang *et al.* 2016b). GFP fusion protein was detected using an anti-GFP mouse monoclonal antibody (Roche) at 1:3,000 dilution. Actin was detected using an anti-actin antibody (ICN Biomedicals Inc.) at a 1:50,000 dilution.

### Microscopy

For microscopy, fresh conidia were inoculated onto sterile glass coverslips overlaid with 1 mL of liquid glucose media with or without 1 M sorbitol. Strains were cultivated on the coverslips at 37° for 14 h before observation. The coverslips with hyphae were gently washed with PBS buffer three times. Differential interference contrast (DIC) and green fluorescent images of the cells were collected with a Zeiss Axio Imager A1 microscope (Zeiss, Jena, Germany).

### RNA extraction for qRT-PCR

The qRT-PCR analysis was performed after growth in liquid glucose or glycerol media for 24 h at 37° in a rotary shaker at the speed of 220 rpm. Total RNA was isolated from the mycelium with TRIzol (Roche) following the manufacturer’s instructions. The genomic DNA digestion and the synthesis of cDNA were performed using HiScript R II Q RT SuperMix for qRT-PCR kit (Vazyme) following the manufacturer’s instruction. qRT-PCR was executed by ABI One-step fast thermocycler (Applied Biosystems) with SYBR Premix Ex TaqTM (TaKaRa). Independent assays were performed with three replicates, and transcript levels were calculated by the comparative threshold cycle (ΔCT) and normalized against the expression of *tubA* mRNA level in *A. fumigatus*. The 2^-ΔΔCT^ was used to determine the changes in mRNA expression. All the qRT-PCR primers and annotations are given in supplementary data (Table S2).

### Statistics

Data are given as means ± SD. The SD was from at least three biological replicates. Statistical significance was estimated with Origin8 using Student’s *t*-test. P-values less than 0.05 were considered statistically significant.

### Data availability

Strains generated in this study are available on request. The authors state that all data necessary for confirming the conclusions presented in article are fully represented within the article and the Supplemental Material. Supplemental material available at Figshare: https://doi.org/10.25387/g3.6224552.

## Results

### gfdA is crucial for colony growth in glucose media

**Figure 1:**
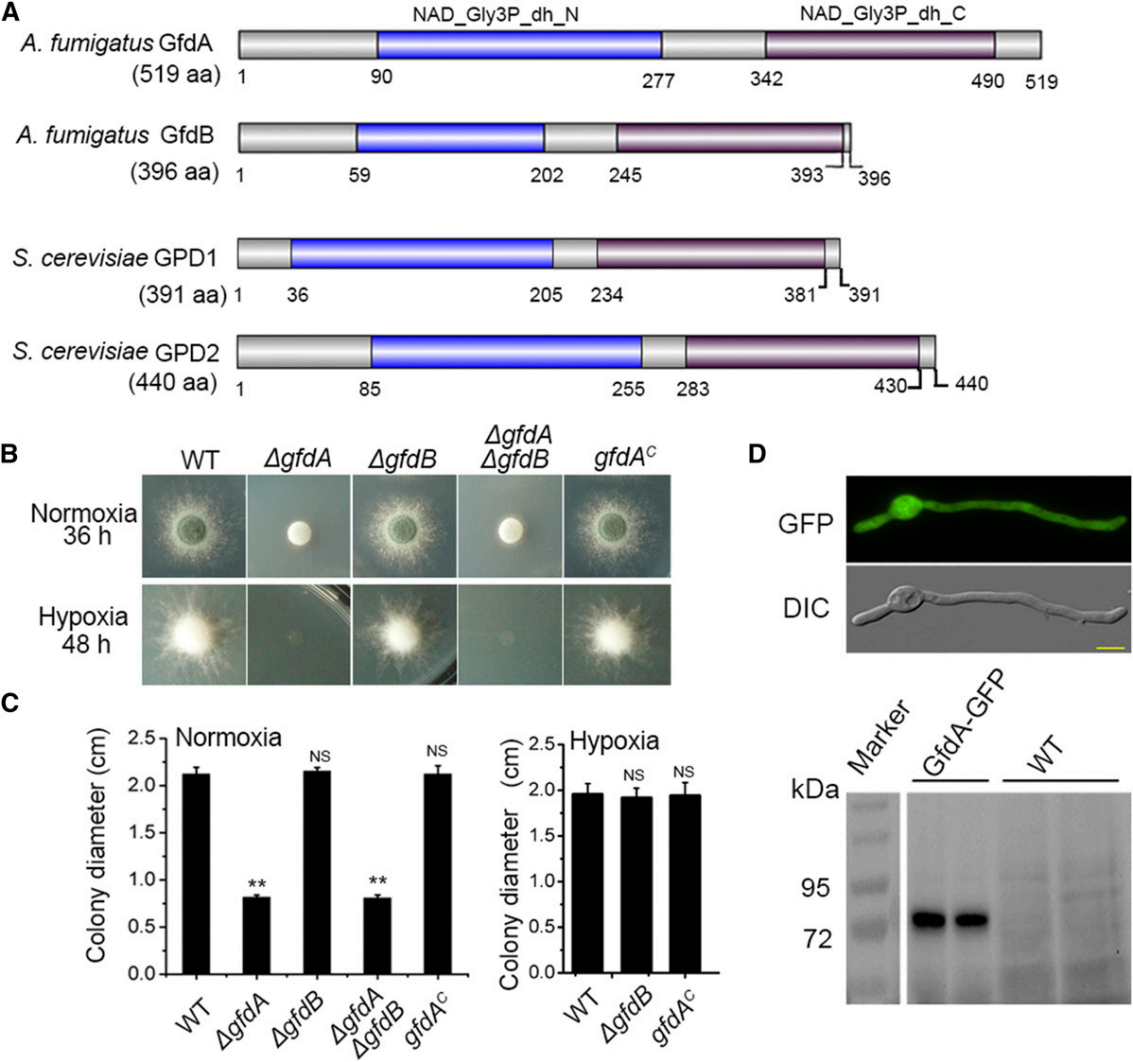
*gfdA* is crucial for colony growth in glucose media under normoxic and hypoxic conditions. (A) Putative protein domain analysis of *A. fumigatus* GfdA, *A. fumigatus* GfdB,* S. cerevisiae* GPD1 and *S. cerevisiae* GPD2. The analysis was derived from SMART (http://smart.embl-heidelberg.de/). The blue rectangle delegates NAD_Gly3p_dh_N domain and the purple rectangle indicates at NAD_Gly3p_dh_C. (B) Colony morphologies of the parental wild-type strain/WT, *ΔgfdA*, *ΔgfdB*, *ΔgfdAΔgfdB*, *gfdA^c^* (*gfdA*-reconstituted strain) in glucose media under normoxic or hypoxic conditions. The 1×10^4^ conidia were inoculated in solid media at 37° for 36 or 48 h. (C) Quantitative data for the diameters of the colonies in related strains. Error bars represent standard deviations from four replicates. Statistical significance was determined by Student’s *t*-test. *P* < 0.05 (*), *P* < 0.01 (**) and *P* > 0.05 (NS). (D) Localization and molecular weight of GfdA-GFP were confirmed by fluorescence microscope and Western blotting. The bar is 5 μm.

To identify the putative homologs in *A. fumigatus*, the *S. cerevisiae* GPD1 and GPD2 sequences were used as queries to perform a BLASTP analysis in the database of *A. fumigatus*. The BLASTP results showed the top homolog candidates are AFUB_002530 (46% identity to GPD1 and 45% identity to GPD2 in protein sequence) and AFUB_024230 (49% identity to GPD1, 48% identity to GPD2), hereafter *gfdA* and *gfdB*, respectively. Furthermore, according to BLASTP analysis, selected species *Schizosaccharomyces pombe*, *Candida albicans*, *Candida glabrata*, and *A. nidulans* also have two G3PDH homologs (Figure S2). Moreover, according to a domain analysis via the SMART software (http://smart.embl-heidelberg.de/), these G3PDH homologs all have the NAD_Gly3p_dh_N domain at their N-terminus and the NAD_Gly3p_dh_C domain at their C-terminus (Figure 1A). To investigate the functions of *gfdA* and *gfdB in A. fumigatus*, we constructed single and double deletions mutants of *gfdA* and *gfdB*. As shown in Figure 1B and 1C, the *gfdA* null mutant (*ΔgfdA*) displayed severe growth defects under both normoxic and hypoxic conditions in solid glucose media when glucose (1%) was used as sole carbon source. In contrast, the *gfdB* single deletion mutant (*ΔgfdB*) showed similar phenotypes to those of the parental wild-type strain in glucose media under the normoxic or hypoxic conditions. Moreover, the *ΔgfdAΔgfdB* double mutant showed a similar growth phenotype as that of *ΔgfdA*, suggesting that *gfdA* plays a predominant role, and that, despite the high levels of homology between their protein sequences, *gfdA* function cannot be replaced by *gfdB* for colony growth. To further characterize function of GfdA, the strain *gfdA-GFP* was generated and showed a similar colony phenotype to its parental wild-type strain in YAG (rich media), indicating that GfdA-GFP was functional (Figure. S3). Florescence microscopy showed that the GfdA-GFP fusion protein was predominantly localized within the cytosol (Figure 1D). Next, a Western blotting experiment was carried out to analyze the molecular mass of GfdA-GFP. Since the GFP protein is about for 27-kD, the relative molecular mass of GfdA was estimated approximately for 56.5 kD, which is consistent with the predicted size of the GfdA protein based on its protein coding sequence. This band was absent in the parental wild-type strain lacking the GFP tag (Figure 1D).

### Overexpressed gfdB is unable to rescue defects of ΔgfdA but has a similar growth enhanced-function as gfdA overexpression in background of wild type

**Figure 2:**
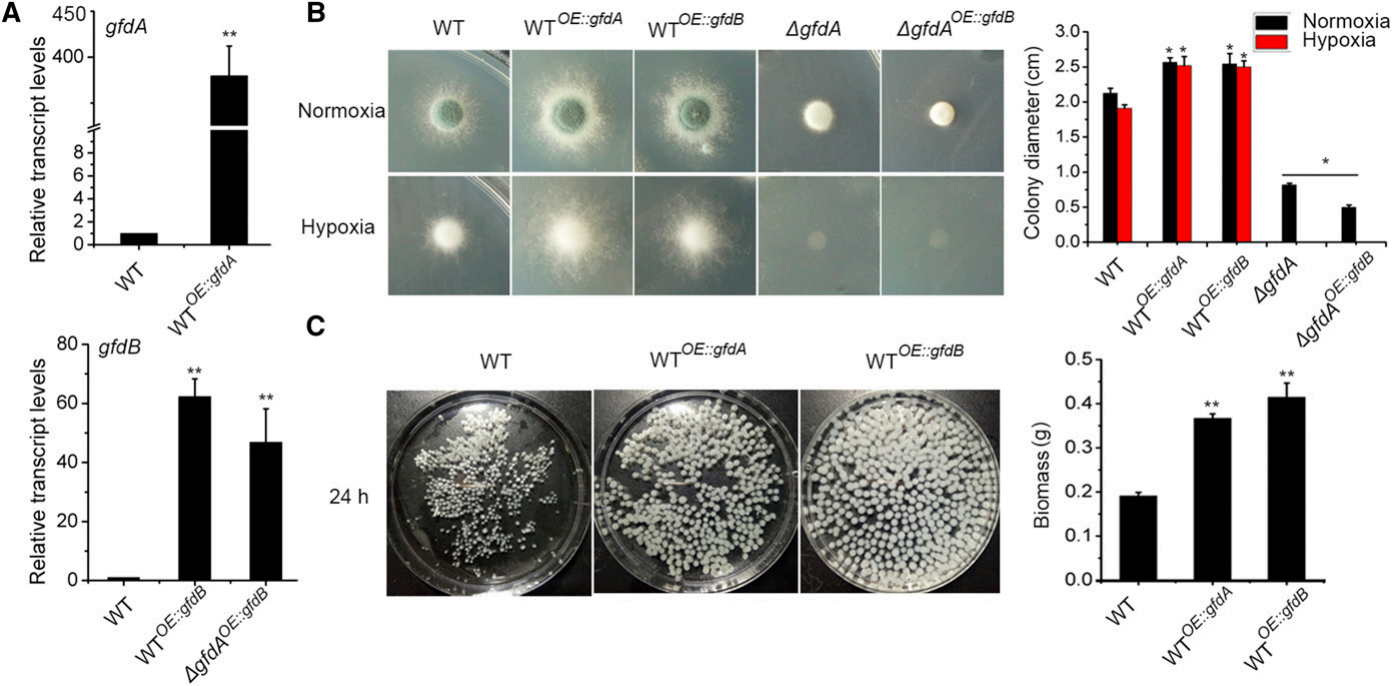
Overexpressed *gfdB* is unable to rescue defects of *ΔgfdA* but has a similar growth enhanced- function with overexpressed *gfdA* in background of wild type. (A) The indicated strains of *A. fumigatus* were incubated in glucose media for 24 h at 37°. Transcript levels of *gfdA* and *gfdB* were determined by qRT-PCR. **P < 0.01. (B) Colony morphologies of WT, WT*^OE^*^::^*^gfdA^*, WT*^OE^*^::^*^gfdB^*, *ΔgfdA*, *ΔgfdA^OE:gfdB^* strains in glucose media under the normoxia (36 h) or hypoxia (48 h) condition at 37°. (C) The analysis for mycelia biomass was performed after cultured for 24 h at 37° for inoculating 5×10^7^ conidia in liquid glucose media in a rotary shaker at the speed of 220 rpm.

In order to further dissect the functions of *gfdA* and *gfdB*, we transformed the full-length ORF sequence of *gfdA* and *gfdB* under the control of the constitutive promoter gpdA (a strong promoter from *A. nidulans*) into the parental wild-type strain, separately, resulting in two overexpression strains, WT*^OE^*^::^*^gfdA^* and WT*^OE^*^::^*^gfdB^*. Taken together with the findings described above and the fact that GfdA and GfdB have the NAD_Gly3p_dh_N domain and the NAD_Gly3p_dh_C domain, we hypothesized that they may have complementary functions in growth and conidiation. Therefore, we overexpressed *gfdB* in a *gfdA* deletion strain (*ΔgfdA^OE^*^::^*^gfdB^*) and confirmed the overexpression of *gfdB* by qRT-PCR (Figure 2A). Unexpectedly, the result showed that not only was *gfdB* overexpression unable to rescue the *ΔgfdA* defects, but that it even exacerbated the defects to some extent (Figure 2B). However, both WT*^OE^*^::^*^gfdA^* and WT*^OE^*^::^*^gfdB^* displayed significantly enhanced production of mycelia biomass in glucose media (Figure 2B and 2C), suggesting that *gfdA* and *gfdB* have a similar functions for promoting the utilization of glucose as carbon source. Taken together, these data suggested that, at least under the conditions tested, *gfdA* plays a dominant role over *gfdB*.

### Glycerol rescues the defects of a gfdA null mutant

**Figure 3:**
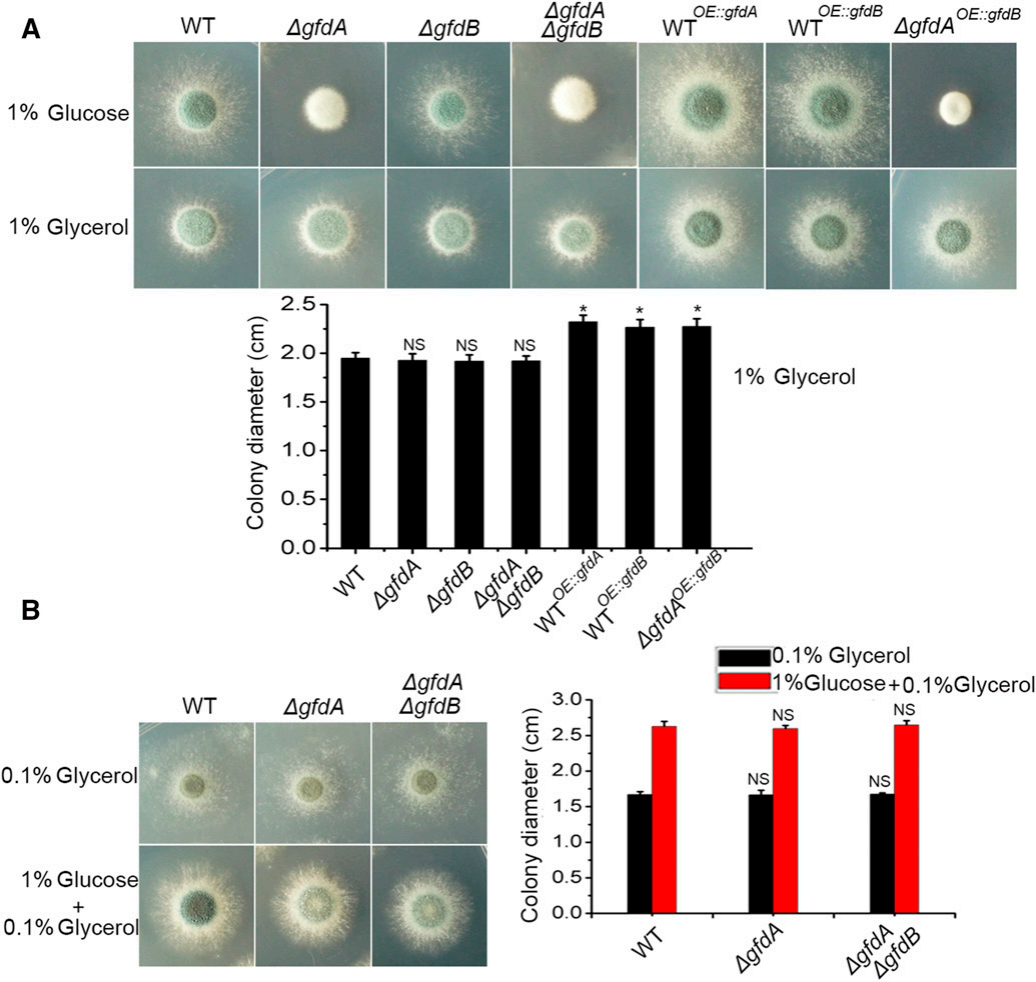
Defects of *gfdA* null mutants were rescued by glycerol. (A) Colony morphologies of WT, *ΔgfdA*, *ΔgfdB*, *ΔgfdAΔgfdB*, WT*^OE^*^::^*^gfdA^*, WT*^OE^*^::^*^gfdB^* and *ΔgfdA^OE:gfdB^* in glucose (1%) media and glycerol (1%) media. The 1×10^4^ conidia were inoculated in solid media for 36 h at 37° (WT as control, *P < 0.05). (B) Colony morphologies of WT, *ΔgfdA* and *ΔgfdAΔgfdB* in the low concentration of glycerol (0.1%) conditions. The 1×10^4^ conidia were inoculated in solid media for 48 h at 37°.

Next, we assayed the phenotypes of *ΔgfdA* and *ΔgfdAΔgfdB* in *A. fumigatus* in glycerol media (glycerol (1%) as the sole carbon source). As shown in Figure 3A, the growth defects of *ΔgfdA* and *ΔgfdAΔgfdB* could be fully restored in glycerol media. Moreover, the three overexpression strains, WT*^OE^*^::^*^gfdA^*, WT*^OE^*^::^*^gfdB^* and *ΔgfdA^OE^*^::^*^gfdB^*, showed faster growth than that of the parental wild-type strain in glycerol media. Finally, a low dose of glycerol (0.1% glycerol) was able to rescue the growth defects of *ΔgfdA* and *ΔgfdAΔgfdB* to the levels of the parental wild-type colonies (Figure 3B). Taken together, these results suggest that defects induced by the *gfdA* deletion may be due to a blockage of glycerol biosynthesis in *A. fumigatus*.

### Overexpression of a predicted glycerol kinase GlcA bypasses the requirement of gfdA in glucose media for colony growth

**Figure 4:**
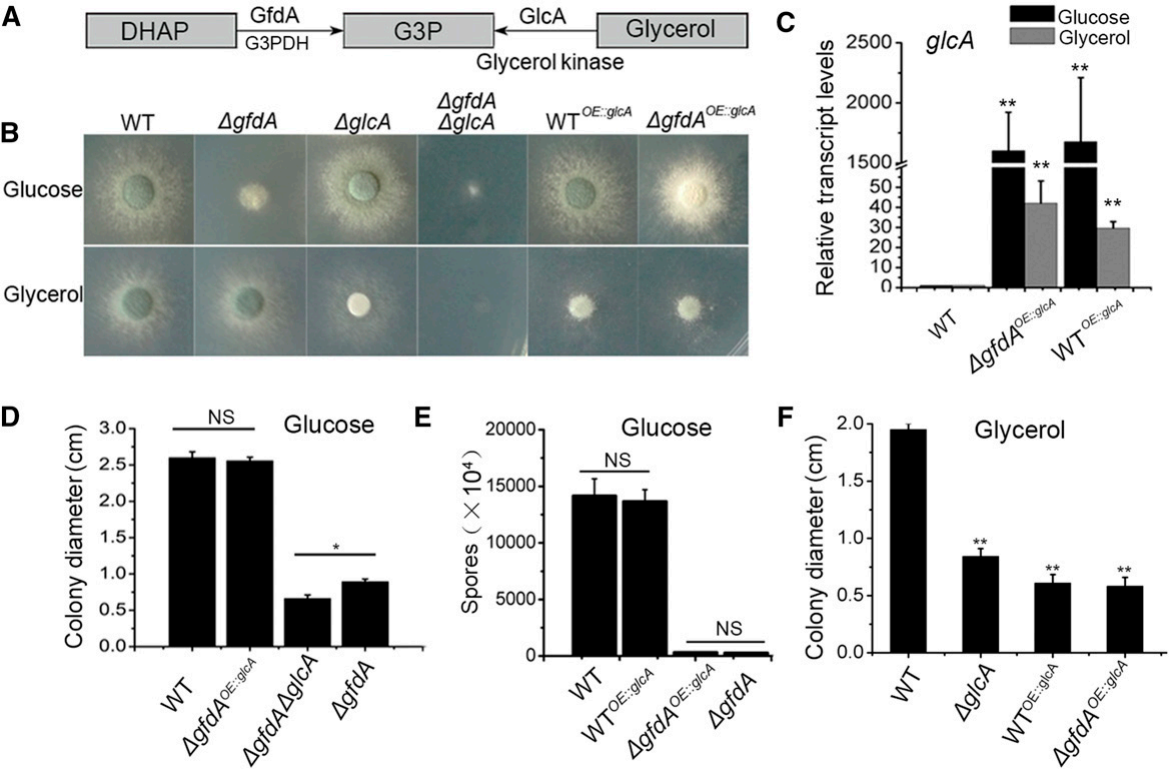
The genetic relationship between *gfdA* and *glcA*. (A) Schematic illustration of the G3P biosynthesis pathway in *Aspergillus*. (B) Phenotypic characterization of WT, *ΔgfdA*, WT*^OE^*^::^*^glcA^*, *ΔgfdA^OE:glcA^*, *ΔglcA*, and *ΔgfdAΔglcA* strains in glucose or glycerol media. The 1×10^3^ conidia were inoculated on solid media for 48 h at 37°. (C) The qRT-PCR analysis was performed after liquid glucose/glycerol media growth for 24 h at 37° in a rotary shaker at the speed of 220 rpm. (D)-(F) The statistical analysis for the diameters and spores of the colonies in related strains.

Next, we investigated whether the defects of growth induced by the loss of *gfdA* were due to lack of glycerol or G3P. G3P can be synthesized by two classical pathways (Figure 4A) (Fillinger *et al.* 2001). The first pathway is catalyzed by an NAD-dependent glycerol 3-phosphate dehydrogenase converting dihydroxyacetone phosphate (DHAP) into G3P The second pathway is catalyzed by a glycerol kinase encoded by *glcA* (AFUB_068560) converting glycerol to G3P (David *et al.* 2006). To further dissect whether *gfdA* and *glcA* had overlapping functions during the glycerol biosynthesis, we knocked out *glcA* in the *ΔgfdA* and the parental wild-type strain backgrounds. The *glcA* null mutant showed defects of growth and conidiation in glycerol media but not in glucose media, suggesting *glcA* is required when glycerol is the sole carbon source, and *gfdA* is required when glucose is the sole carbon source (Figure 4B). Notably, the *ΔgfdAΔglcA* double mutant showed an exacerbation of colony defects in both glucose and glycerol media (Figure 4B). In glucose medium, *ΔgfdAΔglcA* colonies were very small, and colonies were nearly undetectable colonies in the glycerol medium (Figure 4B and 4D). To further verify the functional relationship between *gfdA* and *glcA*, we transformed the full-length ORF sequence of *glcA* under the control of the constitutive promoter gpdA into the *ΔgfdA* and reference strains, resulting in two *glcA*-overexpression strains, *ΔgfdA^OE^*^::^*^glcA^* and WT*^OE^*^::^*^glcA^*. We also confirmed the overexpression of *glcA* by qRT-PCR (Figure 4C). In glucose medium, *ΔgfdA^OE^*^::^*^glcA^* displayed rescued wild-type-like phenotypes for colony growth, but not for conidiation, suggesting that overexpressed *glcA* is able to rescue growth defects associated with loss of *gfdA*. In comparison, WT*^OE^*^::^*^glcA^* still showed wild-type like colony phenotypes (Figure 4B, 4D and 4E). These data suggested that overexpression of *glcA* may result in the production of accumulated G3P, which allowed colonies to bypass the requirement of *gfdA* to produce glycerol use in colony growth, indicating that the growth defects of *gfdA* null mutant might be due to absence of G3P rather than glycerol. In contrast, when glycerol was used as the sole carbon source, *ΔgfdA^OE^*^::^*^glcA^* showed defective phenotypes similar to WT*^OE^*^::^*^glcA^* (Figure 4B and 4F), indicating the overexpression of *glcA* may cause the production of accumulated G3P, which results in growth defects in glycerol media either in the presence or absence of *gfdA*.

### Osmotic stress is capable of bypassing the gfdA requirement for the use of glucose

**Figure 5:**
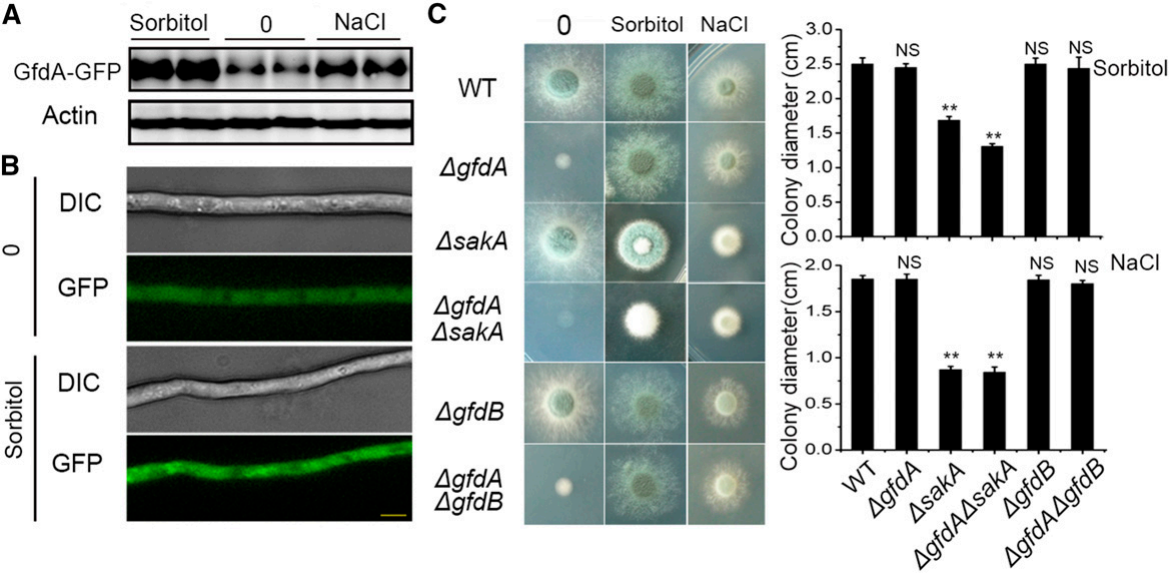
*ΔgfdA* displayed a similar colony growth phenotype to that of its parental wild-type strain under osmotic stress. (A) Western blotting and fluorescence intensity analysis of GfdA-GFP expression in response to sorbitol and NaCl in glucose media. (B) Fluorescence intensity analysis of GfdA-GFP expression in liquid glucose media with or without 1 M sorbitol. The bar is 5 μm. (C) Colony morphologies of WT, *ΔgfdA, ΔsakA*, *ΔgfdAΔsakA*, *ΔgfdB* and *ΔgfdAΔgfdB* in glucose media with or without 1 M sorbitol or NaCl. The 1×10^3^ conidia were inoculated in solid media for 48 h at 37°.

To investigate whether *gfdA* is responsible for the response to osmotic stress, we analyzed the protein expression in the *gfdA-GFP* strain cultured under an osmotic stress condition (1 M sorbitol or NaCl). Our results showed that the expression of GfdA-GFP was clearly increased under the osmotic stress condition, especially by the addition of sorbitol in glucose media (Figure 5A). In addition, microscopy consistently showed that the fluorescence intensity of GfdA-GFP in hyphae treated with sorbitol was greater than that of the non-osmotic stress treatment (Figure 5B). Surprisingly, under osmotic stress, the *ΔgfdA* strain displayed near wild-type colony phenotypes in glucose media (Figure 5C), indicating that the colonies under osmotic stress are capable of bypassing the *gfdA* requirement for the use of glucose. To further explore whether the above recovery of colony growth in *ΔgfdA* under osmotic stress requires the high osmolarity glycerol (HOG) pathway, we first deleted *sakA*, which encodes the protein kinase in the last step of the HOG response pathway (Bruder Nascimento *et al.* 2016), in the *ΔgfdA* and parental wild-type strains. As shown in Figure 5C, under the osmotic stress condition, the *ΔsakA*, *ΔgfdA* double mutant still showed a very severely sick colony phenotype compared to the recovered wild-type colony growth of a *ΔgfdA* mutant. These data indicated that *sakA* is required for bypassing the *gfdA* requirement and suggested that colonies under osmotic stress are capable of bypassing the *gfdA* requirement for the use of glucose in a SakA (HOG)-dependent pathway. In order to explore whether *gfdB* is responsible for the osmotically remediable growth, we tested the phenotypes of *ΔgfdB and ΔgfdAΔgfdB* in osmotic stresses. Both *ΔgfdB* and *ΔgfdAΔgfdB* displayed comparable phenotypes to the parental wild type and to the *ΔgfdA* strain (Figure 5C), suggesting *gfdB* may not play a role in osmotic responses.

## Discussion

Using homology to the *S. cerevisiae* orthologs, we identified two genes, *gfdA* and *gfdB*, that encoding glycerol 3-phosphate dehydrogenase, which catalyzes dihydroxyacetone phosphate to glycerol 3-phosphate (the key substrate of glycerol biosynthesis), in the filamentous fungus *A. fumigatus*, *gfdA*, but not *gfdB*, is necessary for the normal growth of *A. fumigatus* in glucose media. In addition, growth-defective *gfdA* null mutants cannot be rescued by the overexpression of *gfdB*. One possible reason is that *gfdB* may have diverged sufficiently that it has no overlapping function with that of *gfdA*. Another possibility is that *gfdB* might have a different protein localization than that of *gfdA*. Interestingly, in a wild-type background, overexpressing either *gfdA* or *gfdB* is able to significantly enhance biomass production of mycelia. These data suggest that in the presence of *gfdA*, *gfdB* is able to play an additional role in colony growth in glucose media. However, understanding the relationship between *gfdA* and *gfdB* in more detail relationship will require additional work.

The osmolyte glycerol plays an important role in the cellular response to hyperosmositic stresses. Therefore, glycerol 3-phosphate dehydrogenase, a key enzyme in the glycerol biosynthesis pathway, may also play a role in this stress response. Indeed, deletion of the glycerol 3-phosphate dehydrogenase-encoding gene *GPD1* in *S. cerevisia*e conferred hypersensitivity to osmotic stress (Albertyn *et al.* 1994; Ansell *et al.* 1997). However, deleting *gfdA*, the *GPD1* ortholog in *A. fumigatus*, showed no detectable colony phenotype compared to the parental wild-type control strain under hyperosmotic conditions (Figure 5C). This result suggests that, despite their high degree of protein homology, *GPD1* and *gfdA* play different roles in budding yeasts *S. cerevisiae* and filamentous fungi *Aspergillus*. One possible explanation is that *Aspergillus* may use an alternative pathway (DHAP-DHP-Glycerol) to synthesize glycerol in response to hyperosmosis such that it bypasses the requirement of *gfdA* to the extent that the deletion of *gfdA* has no phenotype under osmotic stress (Redkar *et al.* 1995). In contrast, *GPD1* in *S. cerevisiae* is required for the glycerol biosynthesis pathway and the osmotic stress could not bypass the requirement of *GPD1* for colony growth, indicating there may be no alternative pathway to respond to external osmotic stress in the absence of *GPD1*. These data also suggest that *Aspergillus* has developed more a more complex adaptation system for producing glycerol for adaption to hyperosmosis.

In order to survive in a broad range of environmental niches, fungi also possess metabolic pathways that allow them to utilize diverse carbon and nitrogen sources. According to studies on the model fungus A. nidulans and the opportunistic pathogen *A. fumigatus*, the *gfdA* and *glcA* genes play crucial roles in the use of glucose and glycerol, respectively. When glucose is the sole carbon source, it is *gfdA* but not *glcA* that is necessary for normal colony growth and conidiation. In contrast, *glcA* is required for growth in when glycerol is the sole carbon source. Compared to single mutants of *gfdA* and *glcA*, the double mutant *ΔgfdAΔglcA* is very sick in both glucose and glycerol media. Previous studies in *A. nidulans* have demonstrated that the double mutant of *gfdA* and *glcA* produces very little G3P, but contains elevated glycerol content in a glucose media condition (Fillinger *et al.* 2001), which suggests that defects in *ΔgfdA* may result from the blockage of the synthesis of G3P, not glycerol. Our finding that *glcA* overexpression was able to significantly rescue the growth defects of the *gfdA* deletion in glucose media, further supports the hypothesis that *gfdA* is involved in the synthesis of G3P. In addition, the aberrant colony phenotypes produced by the overexpression of *glcA* in a wild-type strain background imply that excessive G3P is toxic for cells. We therefore suggest that fungal cells coordinately produce G3P regulated by GfdA and GlcA to adapt varied carbon resources and survive in different environmental niches.

Therefore, our data demonstrate that G3P plays a crucial role in *Aspergillus* growth so that the reduced production of G3P induced by deletion of *gfdA* may result in blockage of glucose usage. In addition, the decrease of G3P of *ΔgfdA* in *A. fumigatus* may have impact on the integrity and biogenesis of cell wall for colony growth since it displayed the comparable decreased colony growth compared to that of wild type, which may be consistent with that of the *gfdA* deletion in *A. nidulans* (Fillinger *et al.* 2001). In contrast, the excess accumulation of G3P also causes sick colony growth in glycerol media.
